# Different Assay Conditions for Detecting the Production and Release of Heat-Labile and Heat-Stable Toxins in Enterotoxigenic *Escherichia coli* Isolates 

**DOI:** 10.3390/toxins5122384

**Published:** 2013-12-02

**Authors:** Letícia B. Rocha, Christiane Y. Ozaki, Denise S. P. Q. Horton, Caroline A. Menezes, Anderson Silva, Irene Fernandes, Fabio C. Magnoli, Tania M. I. Vaz, Beatriz E. C. Guth, Roxane M. F. Piazza

**Affiliations:** 1 Bacteriology Laboratory, Butantan Institute, São Paulo, SP 05503-900, Brazil; E-Mails: leticia.rocha@butantan.gov.br (L.B.R.); chrisozaki@usp.br (C.Y.O.); caroline.menezes@fleury.com.br (C.A.M.); anderson.dsilva@butantan.gov.br (A.S.); 2 Seroepidemiology and Immunology Laboratory, São Paulo Tropical Medicine Institute, São Paulo, SP 05403-000, Brazil; 3 Fleury-Medicine and Health, São Paulo, SP 04344-903, Brazil; 4 Immunopathology Laboratory, Butantan Institute, São Paulo, SP 05503-900, Brazil; E-Mail: irene.fernandes@butantan.gov.br; 5 Immunochemistry Laboratory, Butantan Institute, São Paulo, SP 05503-900, Brazil; E-Mail: fabio.magnoli@butantan.gov.br; 6 Bacteriology Section, Adolfo Lutz Institute, São Paulo, SP 01246-000, Brazil; E-Mail: vaztmi@gmail.com; 7 Department of Microbiology, Immunology, Parasitology, Escola Paulista de Medicina, Federal University of São Paulo, SP 04923-062, Brazil; E-Mail: bec.guth@unifesp.br

**Keywords:** ETEC, heat-labile toxin, heat-stable toxin, production, release, detection

## Abstract

Enterotoxigenic *Escherichia coli* (ETEC) produce heat-labile (LT) and/or heat-stable enterotoxins (ST). Despite that, the mechanism of action of both toxins are well known, there is great controversy in the literature concerning the *in vitro* production and release of LT and, for ST, no major concerns have been discussed. Furthermore, the majority of published papers describe the use of only one or a few ETEC isolates to define the production and release of these toxins, which hinders the detection of ETEC by phenotypic approaches. Thus, the present study was undertaken to obtain a better understanding of ST and LT toxin production and release under laboratory conditions. Accordingly, a collection of 90 LT-, ST-, and ST/LT-producing ETEC isolates was used to determine a protocol for toxin production and release aimed at ETEC detection. For this, we used previously raised anti-LT antibodies and the anti-ST monoclonal and polyclonal antibodies described herein. The presence of bile salts and the use of certain antibiotics improved ETEC toxin production/release. Triton X-100, as chemical treatment, proved to be an alternative method for toxin release. Consequently, a common protocol that can increase the production and release of LT and ST toxins could facilitate and enhance the sensitivity of diagnostic tests for ETEC using the raised and described antibodies in the present work.

## 1. Introduction

Enterotoxigenic *Escherichia coli* (ETEC), one of the six-diarrheagenic *E. coli* pathotypes (DEC), is responsible for about 300,000 to 500,000 deaths annually in children under five years of age [1]. These organisms are the most frequent cause of traveler’s diarrhea, affecting tourists traveling in endemic areas, as well as the diarrheal pathogen that most commonly afflicts military personnel deployed to endemic areas. In addition, it appears that ETEC contributes substantially to delayed growth and malnutrition, accompanied by repeated bouts of infectious diarrhea, and moreover, malnourished children appear to be at higher risk of acquiring ETEC infections [2,3]. For an effective reduction of these events, preventive measures and easy diagnostic tests are necessary. ETEC causes watery diarrhea after small intestine colonization, mainly through different colonization factors (CFs) and the secretion of heat-labile (LT) and/or heat-stable (ST) enterotoxins that bind to epithelial cell receptors in the intestine. Both CFs and toxins are plasmid-encoded [4].

Heat-stable toxin (ST) is a cysteine-rich peptide synthesized as a pre-pro-peptide of 72 amino acids that are processed during export to produce the mature active toxin of 18 or 19 amino acids [5]. Its C-terminal region is conserved featuring 13 amino acids, of which six are cysteine residues that form three disulfide bonds, necessary for the enterotoxic activity and heat-stable nature of the toxin [6,7]. Once released, ST binds to the extracellular domain of guanylyl cyclase C (GC-C) on the brush border of the intestinal epithelium. These interactions activate the intracellular catalytic domain of guanylyl cyclase, leading to the intracellular accumulation of cGMP, increasing chloride secretion and decreasing sodium absorption [8,9]. 

In contrast to ST, LT is large, oligomeric, with AB_5_ type structure of 84 kDa, and consists of one A subunit and five B subunits [10]. LT is secreted through the outer membrane by a two-step process. In the first step, N-terminal signal peptides of the subunits are cleaved during secretion (sec)-dependent transport across the inner membrane to the periplasm where the monomers assemble into the holotoxin [11,12]. After folding and assembly, the holotoxin is transported across the outer membrane via type II secretion apparatus [13]. In some strains, additional genes, such as *leoA*, coding for a GTP-binding protein [14], and located on a pathogenicity island in the prototype H10407 strain, also modulate LT secretion [15]. Binding of the B subunit to GM1 gangliosides centered in caveolae on the host cell surface triggers endocytosis of the holotoxin [16]. However, how LT is transferred to ganglioside receptors on the surface of intestinal cells has many possible explanations [15,17]. Nevertheless, much of the LT secreted by these organisms under laboratory growth conditions remains associated with outer membrane vesicles, which can enter host cells via lipid raft-dependent endocytosis [18]. Other studies have also suggested that LT and its similar secretion apparatus can assemble or split to one end of the bacterium, thus, allowing ETEC to deliver their toxin at the host cell surface [19,20]. Although there is controversy in the literature concerning the production and release of LT, no such issue has been raised regarding ST. The majority of published papers describe the use of only one or a few ETEC isolates to define the production and release of these toxins [21,22,23], which impairs the detection of ETEC by phenotypic approaches. Thus, the present study was undertaken to achieve a better understanding of ST and LT toxin production and release under laboratory conditions. Accordingly, a collection of LT-, ST- and ST/LT-producing ETEC isolates was used to evaluate different protocols for toxin production and release aimed at ETEC detection. For this detection, we used previously raised anti-LT antibodies [24,25] and anti-ST monoclonal and polyclonal antibodies described herein.

## 2. Results and Discussion

### 2.1. Characterization of ST MAb

**Figure 1:**
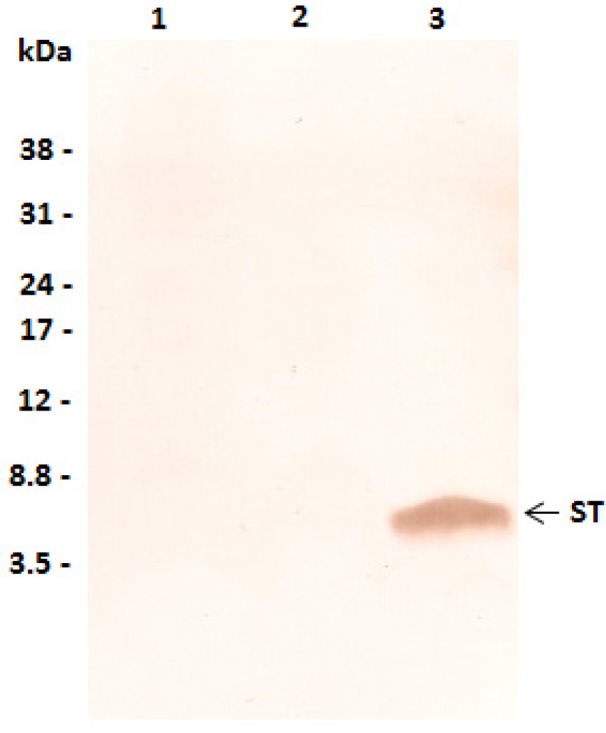
Reactivity of ST MAb by immunoblotting. Bacterial lysates from strains 30 (Lane 1), 127 (Lane 2), and 3321-4 (Lane 3) (3 µg) were separated by SDS-PAGE (10% gel; tricine) and transferred to a PVDF membrane. Each strip was incubated with anti-ST MAb followed by goat anti-mouse IgG peroxidase-conjugate. Immunodetection signals were visualized by addition of DAB/H_2_O_2_. Molecular markers are indicated as kilodaltons (kDa) at the left side of the panel. The arrow indicates the pre-pro-peptide form of ST toxin. ETEC isolates 30 and 127 had spontaneously lost the *estA* gene.

ST MAb was classified as IgG1 and only recognized the ST toxin as determined by immunoblotting (Figure 1) and indirect ELISA. The next step was to investigate the applicability of antibodies in detecting ST in capture ELISA (cELISA). Accordingly, an ST cELISA was standardized using anti-ST MAb in the capture step and an IgG-enriched fraction of rabbit polyclonal anti-ST antibodies as detecting antibody. This system was able to detect as little as 125 ng toxin, showing it to be suitable for our study.

### 2.2. Comparison of Effects of Different Compounds on Production of LT and ST by *E. coli* H10407

**Figure 2:**
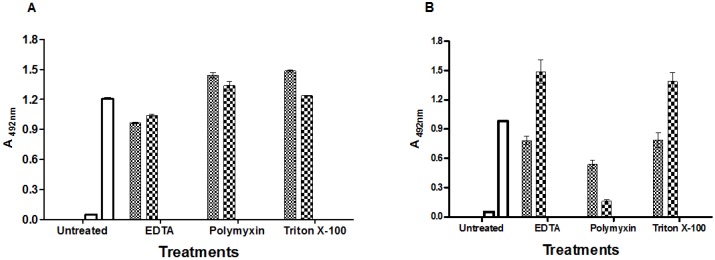
LT (**A**) and ST (**B**) production after chemical treatments. The ETEC H10407 strain was cultivated in EC broth. After 16–18 h, the culture was centrifuged and cell pellets were treated with 0.1 M EDTA or 0.2 mg/mL polymyxin B sulfate (1606 UI/mL) or 2% triton X-100 (small checkered) or not (white bars). The same compounds were also added directly to the culture growth (large checkered) or not (white bars) and then centrifuged. The supernatants treated or not were tested for LT (**A**) or for ST (**B**) by cELISA. The error bars represent the absorbance means and standard errors of duplicates of three independent experiments.

ETEC H10407 is an ST- and LT-producing strain, and was, thus, employed in the evaluation of *E. coli* broth (EC broth). Both toxins were detected by cELISA in supernatants from cells grown in this medium. In order to increase the release of both toxins, the following step was used to test several described treatments using the ETEC H10407 strain. For this strain, toxin release was different; no significant difference was observed in LT release by the addition of polymyxin or triton X-100 to either the cell pellet (*p* = 0.2934) or culture medium (*p* = 0.1545) (Figure 2A). Moreover, there was no significant difference by the use of EDTA or triton X-100 for ST release, either when the pellet was treated (*p* = 0.9354) or in the culture medium (*p* = 0.3692) (Figure 2B). Triton X-100 was the common treatment for the release of both toxins, where pellet treatments released a greater amount of LT (*p* = 0.0013) (Figure 2A), but when this detergent was added directly to the culture medium, the release of ST was significantly increased (*p* < 0.0001) (Figure 2B). 

### 2.3. Effects of Antibiotics on Toxin Production

**Figure 3:**
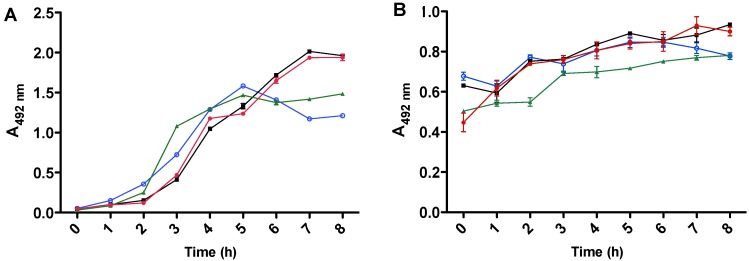
LT (**A**) and ST (**B**) production in presence or absence of antibiotics. The ETEC H10407 strain was cultivated in EC broth containing lincomycin (black line), ciprofloxacin (green line), lincomycin plus ciprofloxacin (red line) or no antibiotic (blue line). The supernatants were tested for LT (**A**) by cELISA or for ST (**B**) by indirect ELISA.

The H10407 strain was cultivated either in the absence or presence of the antibiotic lincomycin or ciprofloxacin and using both antibiotics, we observed that from 6 to 8 h growth, the presence of lincomycin (Figure 3A, black line) increased the production of LT toxin when compared either to its absence (*p* = 0.056) or only ciprofloxacin presence (*p* = 0.079) (Figure 3A, blue and green lines). It was interesting to observe that after 5 h growth, there was a decrease in LT production either in the absence of antibiotic or in ciprofloxacin presence (Figure 3A, green and blue lines). The difference observed for ST production was from 7 to 8 h growth, either employing lincomycin alone (*p* = 0.037), or when both antibiotics were added (*p* = 0.041) (Figure 3B, black and red lines). The production profile observed at 24 h was the same as 8 h of H10407 growth for both toxins in the presence or absence of antibiotics.

### 2.4. Characterization of LT and ST Production/Release in ETEC Strains

On the other hand, in analyzing other ETEC isolates, in addition to H10407, we observed that some of them showed higher toxin production in the presence of lincomycin (data not shown), while for others this production was higher in the presence of ciprofloxacin (data not shown). Also the triton X-100 treatment of the pellet or culture medium had different influences on the release of toxins. Considering these facts, the combination of both antibiotics with EC broth and direct addition of triton X-100 to the culture medium were used to evaluate the toxin production of 90 ETEC isolates.

**Figure 4:**
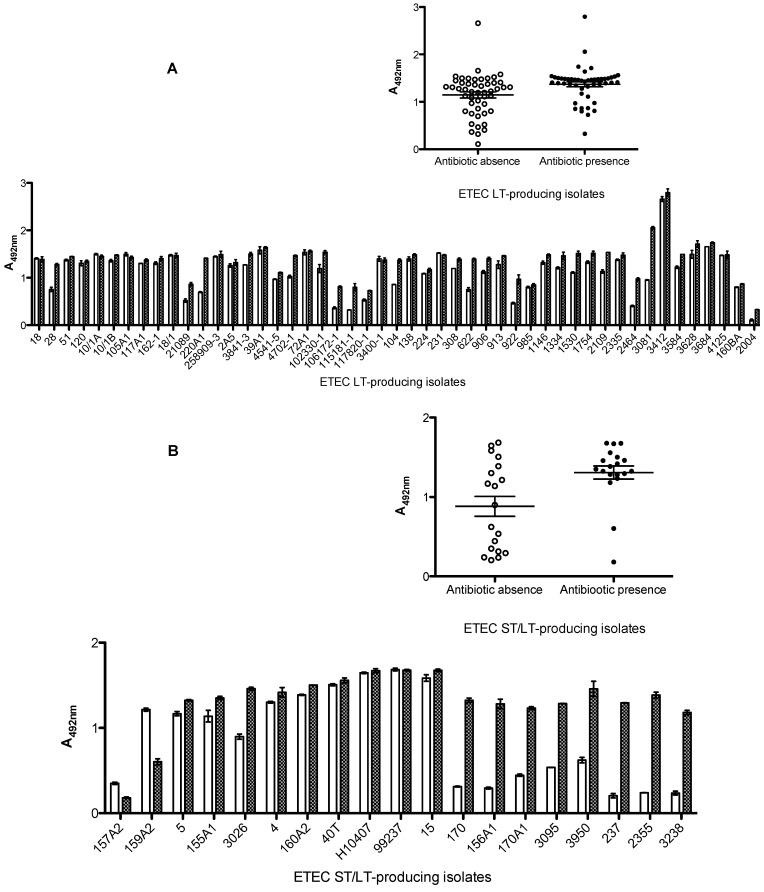
*In vitro* effects of lincomycin and ciprofloxacin on enterotoxigenic *Escherichia coli* isolates. (**A**) LT-producing strains. (**B**) ST/LT-producing strains. The strains were cultivated in EC broth (○/white bars) or EC broth containing lincomycin and ciprofloxacin (●/crosshatched bars), and bacterial growth cultures were treated with 2% triton X-100. Each supernatant was tested for LT by cELISA.

For the LT-producing isolates, we observed that LT production/release was greatly enhanced (*p* = 0.0073) when cells were cultivated in the presence of antibiotics (Figure 4A, insert). All 49 isolates tested showed increased LT production/release level, but variations were observed among the isolates. Notably, for one isolate (2004), LT production/release level was low in the absence of antibiotic, but it was increased three-fold in the presence of antibiotic (Figure 4A).

In analyzing the ST/LT-producing isolates, almost all showed increased LT production/release. Among the 19 tested strains, only two isolates (157A2 and 159A2) showed no increase in LT production/release after antibiotic supplementation in EC broth. In contrast, eight isolates (170, 156A1, 170A1, 3095, 3950, 237, 2355, and 3238) showed at least a three-fold increase in LT production/release with antibiotic addition to EC broth, among them one isolate (170) and three isolates (237, 2355, and 3238) had at least four- and five-fold increased production/release in the presence of antibiotic, respectively (Figure 4B). In addition, for the ST/LT-producing isolates, the addition of antibiotics greatly enhanced LT production/release (*p* = 0.0076) (Figure 4B, insert).

**Figure 5:**
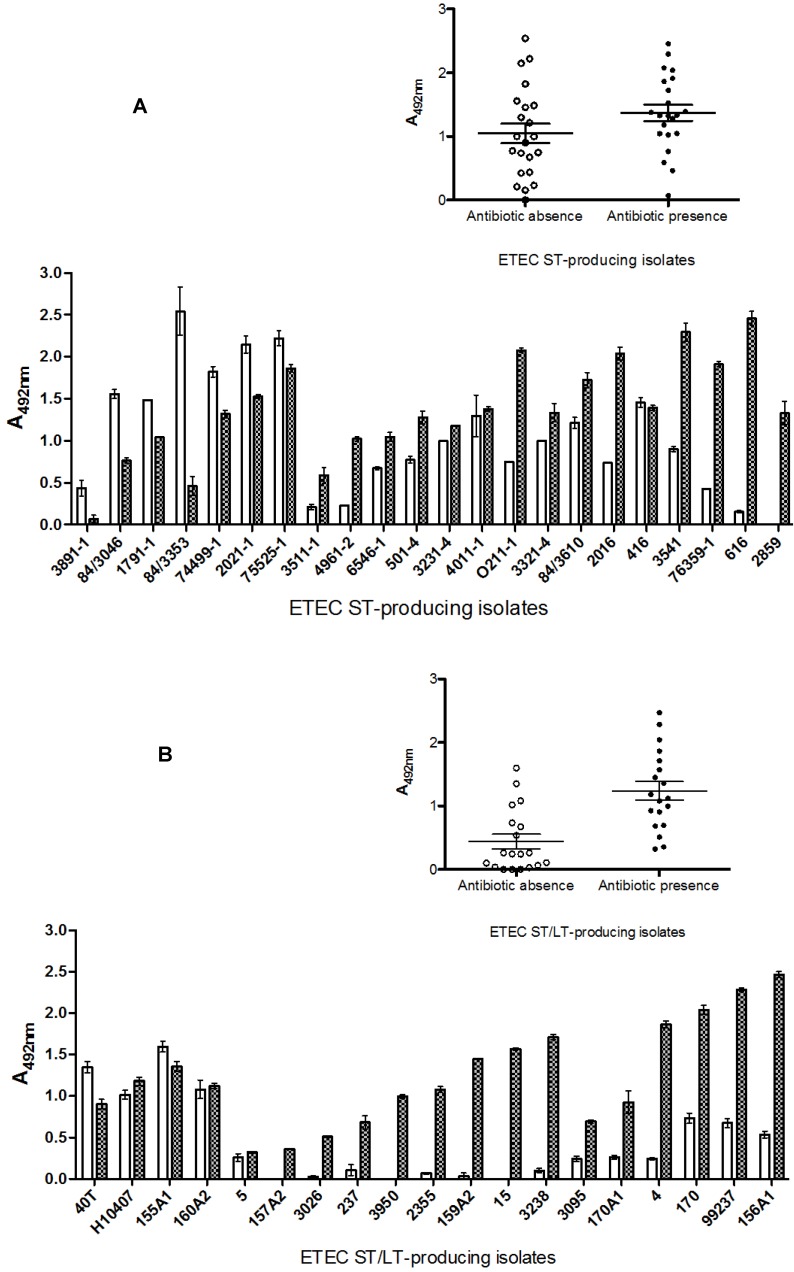
*In vitro* effects of lincomycin and ciprofloxacin on enterotoxigenic *Escherichia coli* isolates. (**A**) ST-producing strains. (**B**) ST/LT-producing strains. The strains were cultivated in EC broth (○/white bars) or EC broth containing lincomycin and ciprofloxacin (●/crosshatched bars), and the bacterial growth cultures were treated with 2% triton X-100. Each supernatant was tested for ST by cELISA.

The influence of both antibiotics together on ST-producing isolates could be seen with increased production/release of ST in 15 ETEC strains, showing at least a two-fold increase. Nevertheless, in one of them (76359-1) there was a more than four-fold increase. In the isolate 616, we observed a 16-fold increase in ST production/release. In addition to the isolate 2859, no ST production was observed in the absence of antibiotic, but in its presence, production/release increased as much as 1000-fold. As noted for LT-producing ETEC strains, seven ST-producing isolates (3891-1, 84/3046, 1791-1, 84/3353, 74499-1, 2021-1, and 75525-1) also showed no change in ST production/release in the presence of antibiotics (Figure 5A). Despite the fact that we observed individual differences between the presence and absence of antibiotics in ST production/release, the absorbance means between groups were not significant (*p* = 0.059) (Figure 5A, insert). 

Almost all ST/LT-producing isolates showed increased production of ST toxin when cultivated in the presence of antibiotics. Among the 14 isolates that showed increased ST production, this was evident only after antibiotic addition in eight (157A2, 3026, 237, 3950, 2355, 159A2, 15, and 3238). Two isolates (40T and 155A1) showed no influence of cultivation with antibiotic on ST production (Figure 5B). However, for the ST/LT-producing isolates, the addition of antibiotics greatly enhanced ST production/release (*p* = 0.0034) (Figure 5B, insert).

### 2.5. Discussion

Among the various conditions described in the literature for the expression of DEC virulence factors, there is no consensus on cultivation and treatment for their production and release, respectively, and the results are often contradictory. In a previous study conducted in our laboratory investigating different culture media to produce Shiga toxin, EC broth was found to be the most suitable medium for this toxin expression [26]. In addition, testing other media with similar composition, but without bile salts, such as Evans medium, Syncase broth and Trypticase Soy Broth, the production/release of LT and ST toxins detected was lower than with EC broth (data not shown). As the effect of EC broth on the expression of ETEC toxins is unknown, and as it was recently demonstrated that *estA* is upregulated by bile salts in the E24377A strain whereas *eltA* is upregulated in H10407 strain [27], one of the objectives of this study was to evaluate, under laboratory conditions, the expression of LT and ST toxins in this medium using a collection of ETEC isolates. Confirming our expectation, EC broth supported the production of both toxins by ETEC strains. Although we did not investigate EC broth without bile salts, this component may stimulate bacterial virulence and may be an environmental signal of the small intestine [27,28].

As part of the LT produced by ETEC, under laboratory growing conditions, remains in the periplasmic space or “associated with outer membrane vesicles” [29], another approach was to investigate bacterial treatment with different chemical compounds to allow the release of intracellular toxin. Several conditions have been described for LT release, and again there is no agreement among studies on the optimal treatment, with results often being conflicting. Polymyxin B has been long used for LT release [21,23,30,31], but some authors have demonstrated that triton X-100 treatment shows a superior performance in LT release [32]. Nevertheless, Lasaro *et al.* [33] found that polymyxin B recovered 25% of LT produced by strain H10407, where it was more effective than triton X-100, which recovered less than 15% of the toxin. The effect of urea has also been described for LT release [34], and in our study, the results of urea treatment showed no reproducibility (data not shown). Although some authors [33,35] consider that “sonic disruption” is the most efficient condition, this method is laborious and impractical in routine use and in dealing with a large collection of bacterial isolates. In our tests, treatment with triton X-100 was the common chemical treatment for the release of both toxins and therefore chosen to be added directly to bacterial culture growth, confirming that production/release of toxins is indeed affected by chemicals *in vitro*.

Next, we investigated the effect of adding antibiotics (ciprofloxacin and lincomycin) to the EC broth on the production of ETEC toxins. It has already been described that the addition of lincomycin to the growth medium causes an increase in LT production of ETEC isolates [22,23,36,37]. As expected, the addition of lincomycin to EC broth increased LT production in the prototype strain (H10407) as well as some LT-positive isolates tested (data not shown). On the other hand, we also observed that the presence of ciprofloxacin privileged LT production in some isolates (data not shown). Yoh *et al.* [37] reported that among several antibiotics tested, either lincomycin or tetracycline alone stimulated the production of LT by ETEC strains, and, as demonstrated by us, the effect of antibiotic on production/release was not similar in all strains. For ST, few data are available; when ST-producing isolates were grown in media with lincomycin, no differences in ST production were detected [38,39,40], as observed with H10407. The increase, though significant, was different between the isolates. However, statistical analyses showed that, depending on the group of isolates, this difference in LT and ST production was higher in strains producing both toxins and LT-producers when cultivated in the presence of antibiotics. Under the same culture conditions, a smaller difference was observed in isolates producing only ST. In fact, according to the literature, no uniformity in the production of toxins by bacterial isolates has been found [41]. A variation of almost 50-fold has been described between LT-positive isolates [42], indicating that the regulation of LT production, as well as ST production, is different in individual ETEC isolates, as demonstrated here with a collection of ETEC isolates. Nevertheless, as far as we know, this is the first time that ST secretion and release characteristics are described.

## 3. Experimental Section

### 3.1. Bacterial Strains

The bacterial isolates used in this study consisted of 90 strains previously defined as ETEC by gene presence including the prototypes H10407 (O78:H11) [43] as the ETEC-producing ST/LT prototype strain and 3321-4 (O153:H45) as the ETEC-producing ST prototype strain [44]. LT-I (49 isolates), ST-I (21 isolates) and LT-I/ST-I (18 isolates) were isolated from different geographic areas as detailed in Table 1 [25,45,46,47,48,49,50,51]. The ETEC isolates (30 and 127), which had spontaneously lost the *estA* gene, were employed as ST toxin negative control [52]. 

**Table 1:** Characteristics of the enterotoxigenic *Escherichia coli* (ETEC) isolates used.

Strain	Serotype	Gene	Toxin expression	Geographic origin/period of isolation
18	O8:H9	*elt*	(LT+)	São Paulo, Brazil, 1994–1996
28	O112:H10	*elt*	(LT+)	São Paulo, Brazil, 1994–1996
51	O62:H19	*elt*	(LT+)	São Paulo, Brazil, 1994–1996
120	O88:H25	*elt*	(LT+)	São Paulo, Brazil, 1994–1996
10/1A	O114:H21	*elt*	(LT+)	Rio de Janeiro, Brazil, 1998
10/1B	O114:H21	*elt*	(LT+)	Rio de Janeiro, Brazil, 1998
105A1	ONT:H4	*elt*	(LT+)	Paraiba, Brazil, 2000–2001
117A1	O6:H16	*elt*	(LT+)	Paraiba, Brazil, 2000–2001
162-1	O88:H25	*elt*	(LT+)	São Paulo, Brazil, 1989–1990
18/1	ONT:HNT	*elt*	(LT+)	Rio de Janeiro, Brazil, 1998
21089	ONT:HNT	*elt*	(LT+)	Rio de Janeiro, Brazil, 1998
220A1	O25:H16	*elt*	(LT+)	Paraiba, Brazil, 2000–2001
258909-3	O128:H1	*elt*	(LT+)	Bangladesh, 1979–1984
2A5	O88:H25	*elt*	(LT+)	Paraiba, Brazil, 2000–2001
3841-3	O88:H-	*elt*	(LT+)	São Paulo, Brazil, 1989–1990
39A1	O109:H19	*elt*	(LT+)	Paraiba, Brazil, 2000–2001
4541-5	O64:H-	*elt*	(LT+)	São Paulo, Brazil, 1989–1990
4702-1	O167:H5	*elt*	(LT+)	São Paulo, Brazil, 1989–1990
72A1	O15:H40	*elt*	(LT+)	Paraiba, Brazil, 2000–2001
102330-1	O45:H16	*elt*	(LT+)	Bahia, Brazil, 2001–2002
106172-1	ONT:H10	*elt*	(LT+)	Bahia, Brazil, 2001–2002
115181-1	O64:H-	*elt*	(LT+)	Bahia, Brazil, 2001–2002
117820-1	O64:H-	*elt*	(LT+)	Bahia, Brazil, 2001–2002
3400-1	ONT:H4	*elt*	(LT+)	Bahia, Brazil, 2001–2002
104	OR:H17	*elt*	(LT+)	Bahia, Brazil, 2003–2004
138	OR:H25	*elt*	(LT+)	Bahia, Brazil, 2003–2004
224	OR:H10	*elt*	(LT+)	Bahia, Brazil, 2003–2004
231	OR:H25	*elt*	(LT+)	Bahia, Brazil, 2003–2004
308	OR:H16	*elt*	(LT+)	Bahia, Brazil, 2003–2004
622	OR:H9	*elt*	(LT+)	Bahia, Brazil, 2003–2004
906	O15:H40	*elt*	(LT+)	Bahia, Brazil, 2003–2004
913	O6:H16	*elt*	(LT+)	Bahia, Brazil, 2003–2004
922	O64:HNT	*elt*	(LT+)	Bahia, Brazil, 2003–2004
985	O166:H15	*elt*	(LT+)	Bahia, Brazil, 2003–2004
1146	OR:H21	*elt*	(LT+)	Bahia, Brazil, 2003–2004
1334	ONT:H16	*elt*	(LT+)	Bahia, Brazil, 2003–2004
1530	OR:H19	*elt*	(LT+)	Bahia, Brazil, 2003–2004
1754	OR:H21	*elt*	(LT+)	Bahia, Brazil, 2003–2004
2109	ONT:H32	*elt*	(LT+)	Bahia, Brazil, 2003–2004
2335	OR:H51	*elt*	(LT+)	Bahia, Brazil, 2003–2004
2464	OR:H10	*elt*	(LT+)	Bahia, Brazil, 2003–2004
3081	OR:H-	*elt*	(LT+)	Bahia, Brazil, 2003–2004
3412	O109:H-	*elt*	(LT+)	Bahia, Brazil, 2003–2004
3584	OR:H32	*elt*	(LT+)	Bahia, Brazil, 2003–2004
3628	ONT:H40	*elt*	(LT+)	Bahia, Brazil, 2003–2004
3684	O133:H25	*elt*	(LT+)	Bahia, Brazil, 2003–2004
4125	O82:H40	*elt*	(LT+)	Bahia, Brazil, 2003–2004
2004	ONT:H-	*elt*	(LT+)	Bahia, Brazil, 2003–2004
160BA	OR:H-	*elt*	(LT+)	Bahia, Brazil, 2003–2004
3321-4	O153:H45	*estA*	(ST+)	São Paulo, Brazil, 1989–1990
616	O25:H16	*estA*	(ST+)	Bahia, Brazil, 2003–2004
2016	O8:H2	*estA*	(ST+)	Bahia, Brazil, 2003–2004
6546-1	ONT:H32	*estA*	(ST+)	Bahia, Brazil, 2001–2002
416	O148:H27	*estA*	(ST+)	Bahia, Brazil, 2003–2004
2859	O78:H27	*estA*	(ST+)	Bahia, Brazil, 2003–2004
3541	ONT:HNT	*estA*	(ST+)	Bahia, Brazil, 2003–2004
74499-1	O23:H15	*estA*	(ST+)	Bahia, Brazil, 2003–2004
76359-1	O166:H15	*estA*	(ST+)	Bahia, Brazil, 2001–2002
1791-1	O29:H21	*estA*	(ST+)	São Paulo, Brazil, 1989–1990
4961-2	O29:H21	*estA*	(ST+)	São Paulo, Brazil, 1989–1990
501-4	O78:H12	*estA*	(ST+)	São Paulo, Brazil, 1989–1990
O211-1	O6:H16	*estA*	(ST+)	São Paulo, Brazil, 1989–1990
3231-4	ONT:H[NT]	*estA*	(ST+)	São Paulo, Brazil, 1989–1990
2021-1	O128ac:H27	*estA*	(ST+)	São Paulo, Brazil, 1989–1990
3511-1	O128ac:H21	*estA*	(ST+)	São Paulo, Brazil, 1989–1990
4011-1	O153:H45	*estA*	(ST+)	São Paulo, Brazil, 1989–1990
3891-1	O153:H45	*estA*	(ST+)	São Paulo, Brazil, 1989–1990
75525-1	O27:H7	*estA*	(ST+)	Bahia, Brazil, 2001–2002
84/3353	O4:H1	*estA*	(ST+)	Germany, 1984
84/3046	O6:HNT	*estA*	(ST+)	Germany, 1984
84/3610	ONT:H5	*estA*	(ST+)	Germany, 1984
H10407	O78:H11	*elt*/*estA*	(LT+ ST+)	Bangladesh, 1973
4	O6:H16	*elt*/*estA*	(LT+ ST+)	São Paulo, Brazil, 1994–1996
5	O6:H16	*elt*/*estA*	(LT+ ST+)	São Paulo, Brazil, 1994–1996
155A1	O6:H16	*elt*/*estA*	(LT+ ST+)	Paraiba, Brazil, 2000–2001
156A1	O6:H16	*elt*/*estA*	(LT+ ST+)	Paraiba, Brazil, 2000–2001
157A2	O6:H16	*elt*/*estA*	(LT+ ST+)	Paraiba, Brazil, 2000–2001
159A2	O6:H16	*elt*/*estA*	(LT+ ST+)	Paraiba, Brazil, 2000–2001
160A2	O6:H16	*elt*/*estA*	(LT+ ST+)	Paraiba, Brazil, 2000–2001
170A1	O6:H16	*elt*/*estA*	(LT+ ST+)	Paraiba, Brazil, 2000–2001
40T	OR:H-	*elt*/*estA*	(LT+ ST+)	São Paulo, Brazil, 1989–1990
99237	O78:H12	*elt*/*estA*	(LT+ ST+)	Rio de Janeiro, Brazil, 1998
237	O6:H16	*elt*/*estA*	(LT+ ST+)	Bahia, Brazil, 2003–2004
2355	OR:H16	*elt*/*estA*	(LT+ ST+)	Bahia, Brazil, 2003–2004
3026	O6:H16	*elt*/*estA*	(LT+ ST+)	Bahia, Brazil, 2003–2004
3095	O6:H16	*elt*/*estA*	(LT+ ST+)	Bahia, Brazil, 2003–2004
3238	O6:H16	*elt*/*estA*	(LT+ ST+)	Bahia, Brazil, 2003–2004
3950	O6:H16	*elt*/*estA*	(LT+ ST+)	Bahia, Brazil, 2003–2004
15	ONT:H19	*elt*/*estA*	(LT+ ST+)	São Paulo, Brazil, 1994–1996
170	O6:H16	*elt*/*estA*	(LT+ ST+)	São Paulo, Brazil, 1994–1996

### 3.2. O:H Identification

Identification of O and H antigens was carried out following standard methods [53] using currently available O (O1–O181) and H (H1–H56) antisera prepared at Instituto Adolfo Lutz with reference strains from *E. coli* and *Klebsiella* (International Reference Centre, Copenhagen, Denmark).

### 3.3. Media, Culture Conditions and Treatments for Toxin Release

Production of ST and LT toxins by the prototype strain (H10407) was evaluated after bacterial cultivation in *E. coli* broth (EC broth; Merck, Rio de Janeiro, Brazil). For culture, just prior to testing, 30 μL of bacteria from the frozen stock (−20 °C) were added to 3 mL of TSB and grown at 37 °C, under stirring conditions (180 rpm), for 18 h. Afterwards, 30 μL of TSB culture were added to tubes containing 3 mL of EC broth and incubated at 37 °C with shaking (250 rpm), either in the absence or presence of 0.1 mg/mL lincomycin and/or 5 ng/mL ciprofloxacin (Cefar, São Paulo, Brazil). Afterwards, cells were removed by centrifugation at 10,000 × *g* and toxin production was measured every hour for 8 h and also after 24 h of incubation by capture or indirect ELISA. 

Conditions for the release of the toxins were evaluated using the prototype ETEC strain cultivated in EC broth for 16–18 h and the cell pellets were treated with: (a) 1 mL of 0.2 mg/mL polymyxin B sulfate (approximately 1606 IU/mL; Sigma-Aldrich Co, St Louis, MO, USA), or (b) 0.1 M EDTA, or (c) 2% triton X-100 (Mallinckrodt Baker, Phillipsburg, NJ, USA), or (d) not treated at 37 °C with shaking (250 rpm) for 1 h. The suspensions (treated or not) were centrifuged at 10,000 × *g* and the supernatants assayed for LT and ST release by capture ELISA. Alternatively, these compounds were added directly to the growth medium and cultures maintained under stirring conditions (250 rpm) at 37 °C for 1 h. After this period, the treated cultures were centrifuged at 10,000 × *g* for 10 min, and the supernatants assayed for LT and ST by capture ELISA. When necessary, the supernatants were stored at −20 °C until use. The best conditions for toxin production and release by the prototype strain were employed to evaluate the 90 ETEC isolates, which were cultivated as described above. 

### 3.4. Rabbit Polyclonal and Mouse Monoclonal (MAb) Anti-LT Antibodies

IgG-enriched fraction of rabbit polyclonal antiserum and IgG2b anti-LT monoclonal antibodies employed were previously described [24,25].

### 3.5. Rabbit Polyclonal and Mouse Monoclonal (MAb) Anti-ST Antibodies

For rabbit immunization, the ST toxin was obtained as follows: the strain 3321-4 was cultivated in 3 L of Staples medium [54] at 37 °C, under stirring conditions (180 rpm), for 18 h. The bacterial supernatant was centrifuged at 10,000 × *g* for 15 min and dialyzed against 40 mM ammonium acetate in dialysis tubing (3.5 kDa MWCO) (Spectrum Laboratories Inc., Rancho Dominguez, CA, USA) at 4 °C for 18 h. After dialysis, the buffer containing protein fractions smaller than 3.5 kDa was lyophilized, and the dried material resuspended in PBS. The protein content was measured using the Micro BCA Protein Assay Kit (Pierce, Rockford, IL, USA) as indicated by the manufacturer. 

A New Zealand White male rabbit (60 days old) was immunized intramuscularly three times at two-week intervals, with a dose of 0.75 mg ST previously coupled to rabbit albumin [55] and adsorbed to 2.5 mg Al^3+^ as adjuvant. Serum was obtained 50 days after immunization. The IgG-enriched fraction of the antiserum was obtained after caprylic acid and ammonium sulfate precipitation [56]. Immune serum reactivity was tested by indirect ELISA.

MAb anti-ST was obtained after immunization of four- to six-week-old female Balb/c mice with 2 µg purified ST. The toxin was purified according to Staples *et al.* [54] previously coupled to mouse albumin [55] and adsorbed to 2.5 mg Al^3+^ as adjuvant. The immunization protocol, hybridoma selection, MAb isotyping and purification were done as already described [24,25,57,58]. The experiments were conducted in agreement with the Ethical Principles in Animal Research, adopted by the Brazilian College of Animal Experimentation, and they were approved by the Ethical Committee for Animal Research of Butantan Institute (469/08).

### 3.6. Evaluation of Anti-ST MAb Reactivity

The reactivity of anti-ST MAb was evaluated by immunoblotting. Bacterial lysates (3321-4, or 30, or 127 strain) were obtained after strain cultivation on colonization factor antigen (CFA) agar plates. Each bacterial growth was removed from the plates and incubated with 1 mg/mL polymyxin B at 37 °C, for 30 min under stirring conditions (200 rpm). After incubation, each bacterial lysate was centrifuged at 10,000 × *g* for 15 min, and the supernatant was stored at −20 °C until use. Through this procedure the toxin was obtained in the pre-pro-peptide form. Three micrograms ST per slot were separated by 10% tricine SDS-PAGE electrophoresis under reducing conditions [59] and transferred to a PVDF membrane (Amersham Biosciences, Little Chalfont, UK) at 150 mA at 4 °C for 18 h. The membrane was cut into 0.5-cm strips and blocked for 1 h with 1% BSA (Sigma-Aldrich, St Louis, MO, USA). The strips were washed three times for 5 min with PBS plus 0.05% Tween-20 (PBS-T) and incubated with anti-ST MAb (30 μg/mL) at 4 °C for 18 h. The strips were washed and incubated for 1 h with goat anti-mouse IgG peroxidase-conjugate (Invitrogen, Carlsbad, CA, USA) diluted 1:5000 in blocking solutions. After washing, immunodetection signals were visualized by addition of diaminobenzidine (DAB) plus H_2_O_2_ (Promega Corporation, Madison, WI, USA) and the reaction was stopped with distilled water. 

### 3.7. Indirect ELISA for ST

For the evaluation of rabbit immune serum, microplates (MaxiSorp microplates, Nunc, Rochester, NY, USA) were coated with ST at 15 μg/mL in 0.05 M sodium carbonate-bicarbonate buffer, pH 9.6, at 4 °C for 18 h. On the other hand, for the evaluation of ST production, microplates were coated at 4 °C for 18 h with supernatant of H10407 strain growth. At each step, plates were washed with PBS-T. Plates were then blocked with 1% BSA in PBS at 37 °C for 30 min. Next, serial dilutions of rabbit serum and IgG-enriched fraction in blocking solution were added and plates incubated at 37 °C for 30 min. Antigen-antibody reaction was detected by addition of goat anti-rabbit IgG peroxidase-conjugate (Sigma-Aldrich, St Louis, MO, USA) diluted 1:5000 in blocking solution at 37 °C for 30 min followed by 0.5 mg/mL *O*-phenylenediamine (OPD; Sigma Aldrich Co, St Louis, MO, USA) plus 0.5 μL/mL hydrogen peroxide in 0.05 M citrate-phosphate buffer, pH 5.0, in the dark at room temperature. The reactions were interrupted after 15 min by addition of 50 μL of 1 M HCl. The absorbance was measured at 492 nm in a Multiskan EX ELISA reader (Labsystems, Milford, MA, USA). At each step, the volume added was 100 μL/well, except in the washing and blocking steps, when the volume was 200 μL/well. All samples were tested in duplicate unless otherwise noted. In contrast, for monoclonal antibodies production, the hybridoma supernatants or dilutions of purified MAb were employed followed by goat anti-mouse IgG peroxidase-conjugate (Invitrogen, Carlsbad, CA, USA) at a dilution of 1:10,000.

### 3.8. Capture ELISA for ST

The production and release of ST by ETEC bacterial isolates was determined by a ST-capture ELISA (c-ELISA) as described below. Microplates (MaxiSorp microplates, Nunc, Rochester, NY, USA) were coated at 4 °C for 18 h with monoclonal anti-ST antibodies at 10 μg/mL in 0.05 M sodium carbonate-bicarbonate buffer, pH 9.6 and blocked with 1% BSA in PBS at 37 °C for 30 min. At each step, plates were washed four times with PBS-T. Next, bacterial supernatants (from culture treated or not) were added and incubated at 37 °C for 2 h. The IgG-enriched fraction of rabbit polyclonal anti-ST antibodies at 50 μg/mL was then added and plates were incubated at 37 °C for 60 min. Goat anti-rabbit IgG peroxidase-conjugate (Sigma-Aldrich, St Louis, MO, USA) diluted 1:5000 in blocking solution was then added followed by further incubation at 37 °C for 30 min. The reactions were developed as described above. The absorbance values represent the mean of duplicates of each strain of three different experiments.

### 3.9. cELISA for LT

The production and release of LT was determined by cELISA as described above with the following exceptions. Capture was done with an IgG-enriched fraction of rabbit polyclonal anti-LT antibodies at 30 μg/mL, and antibody detection was done by incubation with anti-LT MAb (15 μg/mL) followed by goat anti-mouse IgG peroxidase-conjugate diluted 1:10,000 (Invitrogen, Carlsbad, CA, USA). The absorbance values represent the mean of duplicates of each strain of three different experiments.

### 3.10. Statistical Analysis

Absorbance results for the production and release of ST and LT in the absence or presence of antibiotics and after chemical treatments of H10407 strain were evaluated by analysis of variance with GraphPad Prism5^®^; *p <* 0.05 was considered statistically significant.

## 4. Conclusions

The common protocol described in the present work can increase the production and release of LT and ST toxins, which could facilitate and enhance the sensitivity of diagnostic tests for ETEC using the raised and described antibodies herein.
